# Sildenafil Inhibits the Proliferation of Cultured Human Endothelial Cells

**Published:** 2007-06

**Authors:** Ali Erdogan, Doerte Wiebke Luedders, Benedikt Manuel Muenz, Christian Alexander Schaefer, Harald Tillmanns, Johannes Wiecha, Christoph Ruediger Wolfram Kuhlmann

**Affiliations:** 1 *Department of Cardiology and Angiology, Justus-Liebig University of Giessen;*; 2 *Institutes of Physiology and Pathophysiology, Johannes Gutenberg-University of Mainz*

**Keywords:** atherosclerosis, basic fibroblast growth factor, endothelial proliferation, plaque angiogenesis, sildenafil

## Abstract

The proliferation of endothelial cells plays a crucial role in the development of intraplaque angiogenesis (IPA). IPA is a major source of intraplaque hemorrhage and therefore contributes to the destabilization of atherosclerotic plaques. Therefore, the aim of the present study was to examine, whether sildenafil inhibits endothelial cell growth. The proliferation of human endothelial cells derived from umbilical cord veins (HUVEC) was examined on DNA level by measurements of (^3^H)-thymidine incorporation. Cell viability was analyzed using trypan blue staining. The proliferation of cultured human endothelial cells was significantly decreased by 1 μmol/l (-48.4%) and 10 μmol/l (-89.6%) sildenafil (n=10, *p*<0.05). This was not a cytotoxic effect, because cell viability was only reduced at sildenafil concentrations of 50 μmol/l or greater. In addition sildenafil significantly reduced endothelial proliferation induced by bFGF (n=10, *p*<0.05). The presented results demonstrate an antiangiogenic effect of sildenafil that might be useful in the prevention of atherosclerotic plaque vascularization.

## INTRODUCTION

Atherosclerosis is a systemic dysfunctional endothelial, focal occurring, chronic inflammatory, fibro-proliferative, prothrombotic, angiogenic, multifactor disease of the arterial intima caused by the retention of modified low-density lipoproteins, hemodynamic, and reductive-oxidative stress (1-3). Atherosclerotic lesions have a tendency to occur at predictable anatomic sites of the arterial tree. It mainly occurs at bifurcations, side branches, and opposite flow dividers at areas of low endothelial shear stress and turbulent blood flow.

As the eccentric atheroma intima thickens, there is a relative ischemia of the vessel wall, which is a potent inducer of intima angiogensis. The chronic inflammation that runs concurrently serves to magnify this intraplaque angiogenesis, along with endothelial cell dysfunction it contributes to the prothrombotic state of the atherosclerotic plaque. Plaque angiogenesis and intraplaque hemorrhage may be associated with unstable vulnerable plaques and contribute to plaque destabilization (1). Kumamoto *et al*. (4) were able to reveal that intimal-medial neovascularization is closely associated with the inflammatory reaction within the plaque, established early in the atherosclerotic process. The vulnerability of the atherosclerotic plaque results from the fact that these microvessels are very fragile and prone to leak and rupture-creating intraplaque hemorrhages (IPH). These IPH as an injury or angiogenic stimulus for the progression of increasing numbers of microvessels within the atherosclerotic vessel wall. Vessel wall remodeling including angiogenesis is important in the development of the vulnerable unstable atherosclerotic plaque and an important factor in the development of acute coronary syndromes. As the numbers of intraplaque microvessels increases, the numbers of IPH increase as a result and contribute to the instability of the atherosclerotic plaque (4, 5). For this reason, the development of antiangiogenic therapeutic approaches might be beneficial in the prevention of plaque rupture.

Since endothelial proliferation is a prerequisite for angiogenesis, the aim of the present study was to examine whether the phosphodiesterase type 5 (PDE5) inhibitor sildenafil affects the proliferation of human endothelial cells *in vitro.*

## MATERIALS AND METHODS

### Cell isolation and culture

Human endothelial cells derived from the umbilical cord veins (HUVEC) were isolated by a collagenase digestion procedure as described before (6). Cells were grown in endothelial cell basal medium (EBM; PromoCell, Heidelberg, Germany) with the addition of 10% fetal calf serum (FCS; PAA, Linz, Austria). The following substances were added to the culture medium: 0.4% endothelial growth supplement/heparin, epidermal growth factor 0.1 ng/ml, hydrocortisone 1 μg/ml, basic fibroblast factor 1 ng/ml, gentamicin 50 μg/ml (PromoCell, Heidelberg, Germany). Culture medium was changed every 48 h. All experiments were carried out using endothelial cells from subcultures one to five.

### Cell viability

To test whether sildenafil (Pfizer, Sandwich, U.K.) has cytotoxic effects on HUVEC, cell viability was examined using trypan blue staining. Cells were cultured in 12 well plates until they had reached confluence. The EBM containing the above mentioned supplements were changed to serum free EBM containing hydrocortisone and gentamicin only (SFEBM). After 24 hours medium was exchanged again and HUVEC were kept in EBM containing 2% FCS and various concentrations of sildenafil (0.001 μmol/l - 500 μmol/l). The next day cells were stained with trypan blue for 5 minutes at 37°C. Medium was collected and the cells were washed two times with HBSS (PAA, Linz, Austria). Afterwards cells were trypsinized (Trypsin-EDTA; Sigma, Deisenhofen, Germany) and transferred to the previous collected medium. After centrifugation cells were washed three times using HBSS. Cell pellets were resuspended and counted four times in a Neubauer chamber. The number of viable cells was set in relation to the total number of cells counted.

### Cell proliferation

Endothelial cell proliferation was detected on DNA-level as described before (7). HUVEC were seeded at a density of 5000 cells/cm^2^ and cultured in EBM containing all supplements for 24 hours. Then medium was changed to SFEBM for another 24 hours. Cell stimulation was performed in EBM containing 2% FCS and the following substances: sildenafil (1-10 μmol/l); basic fibroblast growth factor (50 ng/ml; bFGF; Peprotech, London, U.K.). After 20 hours 1 μCi/ml of (^3^H)-thymidine (Amersham, Freiburg, Germany) was added to each well. 4 hours later cells were washed with ice-cold PBS three times, fixed with 100% cold methanol for 15 min at 4°C, precipitated with 10% cold trichloroacetic acid for 15 min at 4°C, washed with water three times, and lysed with 200 μl of 0.1 N NaOH for 30 min at room temperature. (^3^H)-Thymidine incorporation was measured in scintillation solution using a β-counter (Canberra-Packard, Dreieich, Germany). The activity of control cells was defined as 100%, and the activity of treated cells was set in relation to the activity of the control cells.

### Statistical analysis

The results of all experiments are demonstrated as mean values ± SEM. Statistical significance between groups was determined by ANOVA followed by post hoc Tukey test (SPSS for windows 11.0).

## RESULTS

### Dose-dependent cytotoxic effects of sildenafil

**Figure 1:**
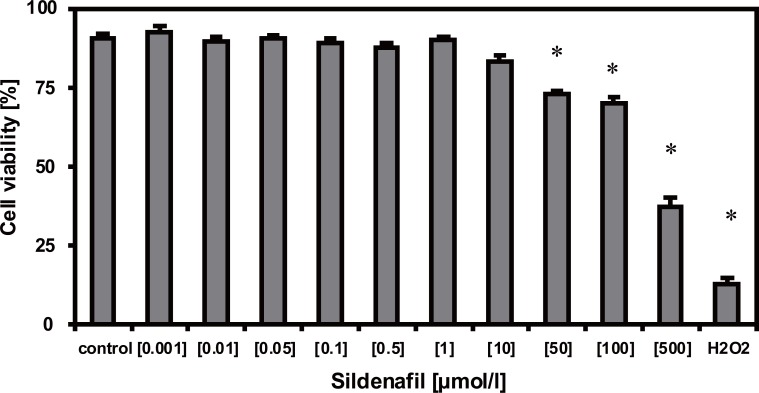
Sildenafil dose-dependently exerts cytotoxic effects. Cell viability of HUVEC was examined using trypan blue staining. The number of viable cells was set in relation to the total cell number and is expressed as cell viability in %. Data represent mean values ± SEM. Statistical significant changes induced by the various sildenafil concentrations (0.001-500 μmol/l) compared to the untreated control group are indicated (n=10, *p<0.05 vs. control). Cells treated with 0.01% H_2_O_2_ served as positive control.

Sildenafil at concentrations greater than 10 μmol/l exerted cytotoxic effects on HUVEC. As demonstrated in Figure 1 sildenafil significantly decreased cell viability to 72.9% (50 μmol/l), 69.8% (100 μmol/l), and 37.1% (500 μmol/l) (n=10, *p*<0.05). Therefore in the following experiments sildenafil was applied at a concentration of 1 and 10 μmol/l, respectively.

### Sildenafil prevents endothelial proliferation

**Figure 2:**
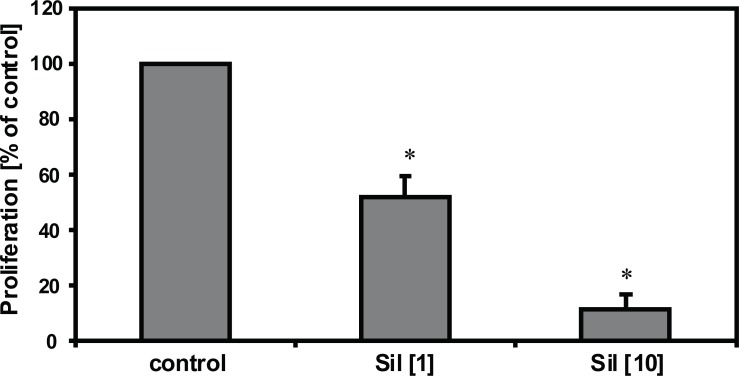
Inhibition of endothelial proliferation by sildenafil. HUVEC proliferation was detected using (^3^H)-thymidine incorporation. Sildenafil (1 and 10 μmol/l) significantly reduced endothelial proliferation (n=10, *p<0.05 vs. control). Results are expressed as % of prolifearation compared to control.

**Figure 3:**
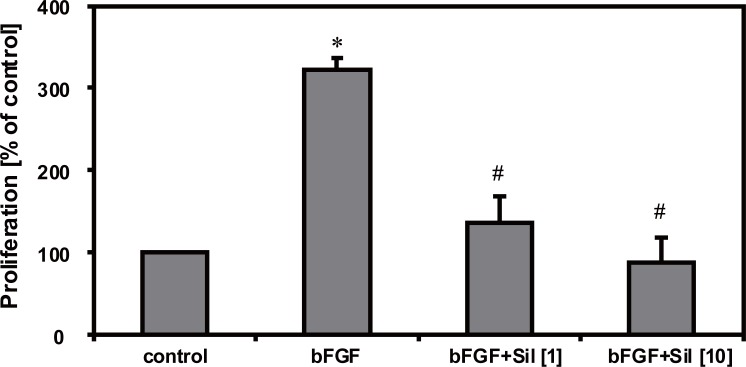
Sildenafil inhibits bFGF-induced HUVEC proliferation. HUVEC proliferation was detected using (^3^H)-thymidine incorporation. BFGF (50 ng/ml) caused a significant increase of endothelial proliferation, that was blocked by sildenafil (1 and 10 μmol/l) (n=10, *p<0.05 vs. control, ^#^*p*<0.05 vs. bFGF). Results are expressed as mean values ± SEM in % of proliferation compared to control.

Endothelial proliferation was significantly decreased by 1 and 10 μmol/l sildenafil (n=10, *p*<0.05). In detail, sildenafil reduced the DNA-synthesis to 51.6% (1 μmol/l), and 11.4% (10 μmol/l). These results are shown in Figure 2. In order to examine whether the antiproliferative effect of sildenafil is also able to affect endothelial cell growth induced by a strong mitogen, the experiments were repeated with the addition of a high concentration (50 ng/ml) of bFGF. As expected, the mitogenic response of the cultured endothelial cells was significantly increased by 321.7% if 50 ng/ml bFGF was supplemented (n=10, *p*<0.05). Interestingly bFGF-induced HUVEC proliferation was significantly, strongly decreased by sildenafil as demonstrated in Figure 3.

## DISCUSSION

The present study was performed to examine whether sildenafil might be a pharmacological substance that is able to prevent endothelial proliferation. The results of the experiments demonstrate for the first time an antiproliferative effect of sildenafil on human endothelial cells *in vitro*, and that this inhibition of endothelial cell growth was not due to a cytotoxic effect. These findings present new therapeutically aspects for sildenafil that are far beyond the use of sildenafil as vasodilator in pulmonary hypertension or erectile dysfuntion. Since sildenafil increases cGMP levels by blocking the PDE5, it is tempting to speculate that mechanisms similar to those observed in vascular smooth muscle cells exist in endothelial cells (8). Since sildenafil has been demonstrated to induce nitric oxide (NO) dependent mechanisms (9) another possible explanation could be that NO reduces endothelial proliferation. This would be in line with the findings of other working groups, which have already demonstrated an antiproliferative effect on endothelial cells mediated by nitric oxide (10, 11). It has to be mentioned, that Heller *et al*. concluded that the antiproliferative effect of nitric oxide was independent of cGMP (10), which would exclude a cGMP-dependent mechanism involving the protein kinase G. Further experiments have to be performed to analyze the exact mechanisms that are involved in sildenafil-induced inhibition of endothelial cell growth. Nevertheless the results of the present study might be of relevance for the treatment of patients suffering from atherosclerosis. Increased angiogenesis has been reported in both coronary artery- and carotid artery- atherosclerotic plaques (12-14). Up to now there are no clinical studies to examine the possible beneficial effects of an antiangiogenic therapy in atheroscelrosis treatment. The most common animal model to examine atherosclerosis is the apolipoprotein E deficient mice model. The study of Moulton *et al*. (15) demonstrated that inhibition of angiogenesis significantly decreases atherosclerosis in this animal model, indicating that there might be a clinical indication for antiangiogenic drugs in the therapy of atherosclerosis. Sildenafil might be used as an antiangiogenic drug to prevent pathological states of angiogenesis as they are found in atherosclerosis, rheumatoid arthritis and tumor vascularization.
